# Supplementary material for: Genome-Wide Association Analysis of Growth Traits in Hu Sheep
Source: Genes (Basel). 2024 Dec 20;15(12):1637. doi: 10.3390/genes15121637 (PMC11675594; doi:10.3390/genes15121637)
Supplement: Supplementary file 1 [file genes-15-01637-s001.zip › Supplementary Files/Figure S1.docx]

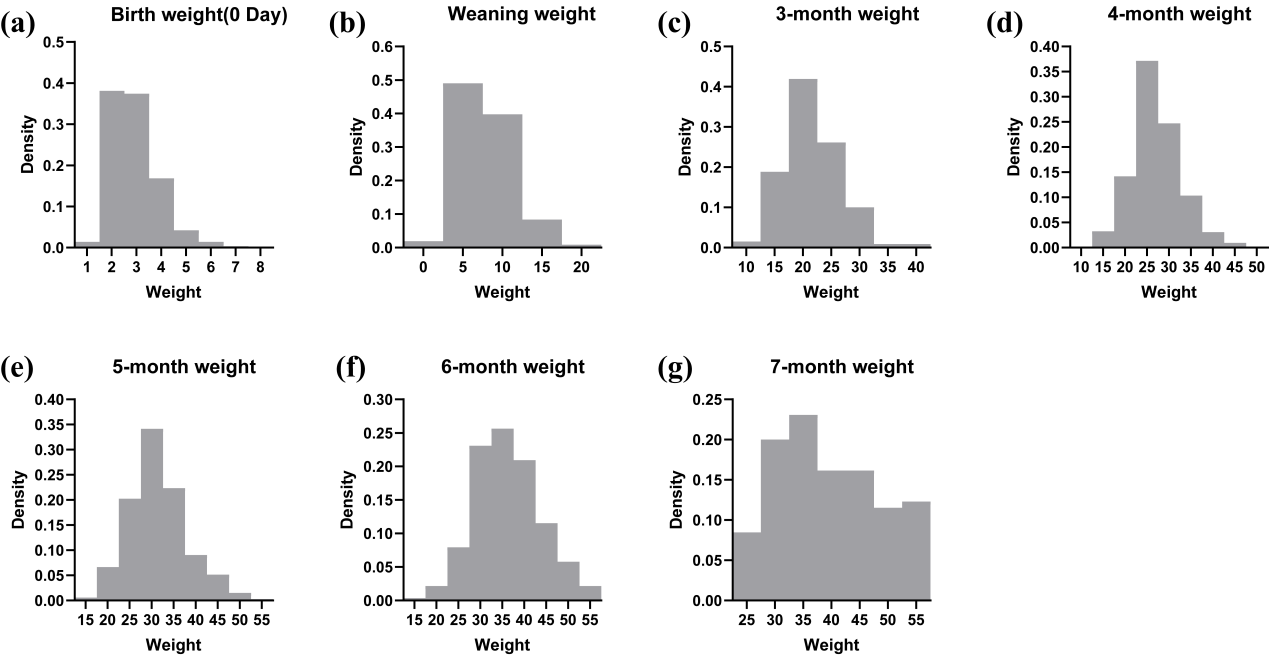


Figure S1 Distribution of body weight data at different ages before outlier removal of Hu sheep. (a) Birth weight; (b) Weaning weight; (c) 3-month weight; (d) 4-month weight; (e) 5-month weight; (f) 6-month weight; (g) 7-month weight.
